# Therapeutic Targets of Bufalin on Renal Carcinoma and Mechanisms: Experimental Validation of Network Pharmacology Analysis

**DOI:** 10.1155/2022/5469795

**Published:** 2022-01-28

**Authors:** Lei Zhang, Yi-Ming Pan, Lu-Yao Wang, Wan-Zhu Zhao, Shao-Cheng Yang, Xue-Fang Sun, Xin Guan, Bing Yan

**Affiliations:** ^1^College of Life Science and Technology, Mudanjiang Normal University, Mudanjiang 157011, China; ^2^School of Public Health, Xinjiang Medical University, Urumqi 830017, China

## Abstract

The possible targets underlying the activity of bufalin on renal cell carcinoma (RCC) were investigated using network pharmacology and experimental approaches. PharmMapper and other databases were explored for predicting the bufalin targets and RCC-related targets. Finally, the enriched pathways and the targets were analyzed by the Kyoto Encyclopedia of Genes and Genomes (KEGG) and Gene Ontology (GO) pathway enrichment analyses. Furthermore, in vitro cell experiments were used to verify bufalin activation of AKT and MAPK signaling pathways in human mesangial cells. The therapeutic targets related to bufalin were identified via 35 intersecting targets. GO analysis identified 29 molecular functions, 16 cellular components, and 91 biological processes. KEGG pathway annotation identified 15 signal transduction pathways and 4 tumor-related pathways.

## 1. Introduction 

Bufalin (C_24_H_34_O_4_), a major monomer component of toad venom, is extracted from toads for use in traditional Chinese medicine. It is derived from traditional Chinese medicine and includes a single component and is milder than Western medicines [1]. Bufalin has received increasing attention of researchers and clinicians due to its anticancer, heart-strengthening, analgesic, and other effects [2, 3]. Bufalin has been shown to have antitumor efficacy against a variety of tumors, including mouth cancer, esophageal cancer, and bladder cancer [4–6]. Bufalin's anticancer action is distinguished by its low dose and highly selective nature. At the molecular level, studies employing bufalin have investigated the occurrence and growth of cancer cells, cell cycle, apoptosis, and gene expression. This study aimed to look into the biological signaling pathways and the predicted therapeutic targets of bufalin for treating RCC as well as providing experimental data and bioinformatics for basic clinical research on RCC treatment, using a scientific strategy based on network pharmacology.

## 2. Materials and Methods

### 2.1. The Target of Bufalin Resistance to RCC Was Obtained and Predicted

**Figure 1:**
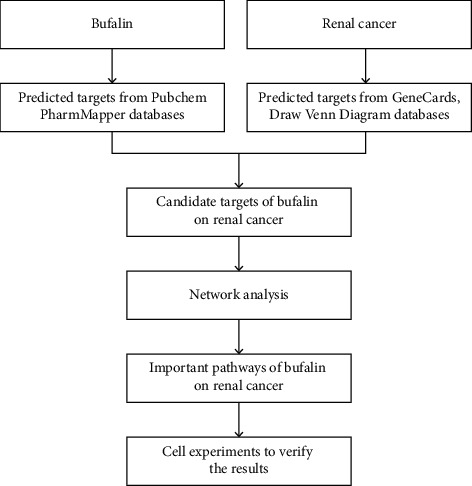
The analysis design of bufalin on renal carcinoma is depicted in a flowchart.

The PubChem website and PharmMapper server were used to predict potential targets for all the predicted targets of bufalin [7]. Additionally, the Draw Venn Diagram website (https://Bioinformatics.psb.ugent.be/webtools/Venn/) in the GeneCards database (https://www.Genecards.org/) [8] was used for intersection comparison, and the possible anti-RCC targets of bufalin were acquired from the GeneCards database (Figure 1).

### 2.2. PPI Network Construction and Topological Analysis of Bufalin

The anti-RCC protein-protein interaction (PPI) network was generated using the Cytoscape 3.7.2 software, and the target and target functional proteins were collected using the online String program (https://string-db.Org/). The maximum degree of freedom in the PPI network of topological parameters of the average sum was analyzed by using Cytoscape using the landscape analysis approach.

### 2.3. GO Enrichment Analysis and KEGG Pathway Annotation

The biological information annotation database (https://David.NCIFCRF.gov/, David) [9] was used to conduct GO and KEGG analyses, and GO enrichment analysis covered biological processes, molecular function (MF), and cellular components (CC). The analysis results were downloaded, and the *P* value <0.05 was set for further screening analysis and mapping.

### 2.4. Replication Experiment

#### 2.4.1. Materials

Bufalin was purchased from Beijing Solaibao Technology Co. Ltd. (China). Caki-1 cells were donated by Harbin Institute of Technology cell laboratory, and human normal liver cells were retained by the Biology Department of Mudanjiang Normal University. All cell culture reagents were from HyClone (USA). The cell cycle detection kit and FITC kit were purchased from Invitrogen (Nanjing, China). PI3K and Akt1/2/3 were purchased from Santa (Wuhan, China). *β*-Actin was purchased from Boaosen (Wuhan, China).

#### 2.4.2. Cytotoxicity Assay

Human normal liver cells were cultured in an incubator with 5% concentration of CO_2_, and the cells were inoculated into a 96-well culture plate at the rate of 1000 cells per well. The cytotoxic effects were assessed using the MTT assay. Experimental groups were treated with bufalin at the final concentration of 0.1, 1, and 10 *μ*mol/L for 24 hours. All of the tests were carried out in triplicate, and the results were expressed as inhibition rate.

The cytotoxic effects were assessed using the MTT assay. Bufalin was added to the experimental group at final concentrations of 1, 10, 20, 50, 100, and 1000 nmol/L, while the conventional medium was added to the control group. The durations for the treatment were 12 h, 24 h, and 48 h. All of the tests were carried out in triplicate, and the results were expressed as the mean IC_50_ values.

### 2.5. Annexin V/PI Staining for Apoptotic Cell Death

In brief, bufalin was applied to Caki-1 cells for 12 and 24 hours at varying concentrations (5, 10, 20, 50, 100, and 200 nmol/L) following the method reported by Ni et al. [10]. The cells were digested by using trypsin without EDTA and collected. The collected cell density was adjusted to 1 × 10^6^ cells/mL using 1x binding buffer. Annexin V-FITC 15 *µ*L and 5 *µ*L PI were added to cell suspension (100 *µ*L) and gently mixed. After staining, the cells were rinsed with PBS (containing 0.5% FBS). Finally, the cells were resuspended with 400 *µ*L binding buffer for flow cytometry analysis.

#### 2.5.1. Cell Cycle Analysis

Various concentrations of bufalin (1, 5, 10, 20, and 50 nmol/L) were added to the cells for 24 h, following the method reported by Ni et al. [11]. After culture, the sample was digested with 0.25% trypsin and 0.02% EDTA, and the cells were collected. The cells were cleaned with PBS twice and centrifuged at 4°C for 5 min at 1000 rpm, and the supernatant was discarded. The mixture was incubated at 37°C for 30 min at dark, which contains 10 μL ribonuclease (RNaseA, 25 μg/mL) and 10 μL freshly prepared PI staining solution (50 μg/mL). Flow cytometry was used for analysis.

### 2.6. Western Blotting Analysis

Cells were treated with bufalin at concentrations of 10, 100, 500, and 1000 nmol/L and cultured for 24 h, following the method reported by Ni et al. [11]. A total of 30 *μ*g protein from each well was separated by 8% (or 10%) SDS-PAGE and transferred to polyvinylidene difluoride (PVDF) membranes. The membranes were blocked at room temperature for 1 h with 5% skim milk in tris-buffered saline with 0.1% Tween 20 (TBST), followed by incubation with the primary antibody diluted with 5% skim milk in TBST at 4°C overnight, and then incubated with the secondary antibody and detected using the ECL chemiluminescent system. Immune complexes were formed by incubation with the following antibodies: anti-MAPK, anti-PI3K, and anti-AKT. Loading differences were normalized using a monoclonal *β*-actin antibody.

### 2.7. Statistical Analysis

The SPSS 13.0 statistical software was used for the data analysis. A *P* value of less than 0.05 was considered statistically significant.

## 3. Results

### 3.1. Target Network Analysis

The top 100 target proteins were finally obtained in the PharmMapper server, and 73 target proteins with a *Z*-score >0 were selected, which were then imported into the UniProt database and corrected into gene symbol and gene ID. Furthermore, 30 goals were listed based on the *Z*-score ranking (Table 1).

### 3.2. Bufalin-Related Targets for the Treatment of RCC

**Figure 2:**
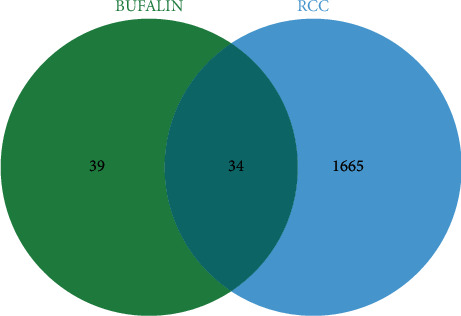
Venn diagram of bufalin targets and RCC-related pathogenesis targets.

To obtain renal cancer-related disease targets, 1699 RCC-related targets were obtained using the GeneCards database. The intersection comparison between bufalin targets and RCC-related targets was conducted, and the Venn diagram identified 34 possible targets with connections to RCC and bufalin (Figure 2).

### 3.3. Bufalin-RCC Network

**Figure 3:**
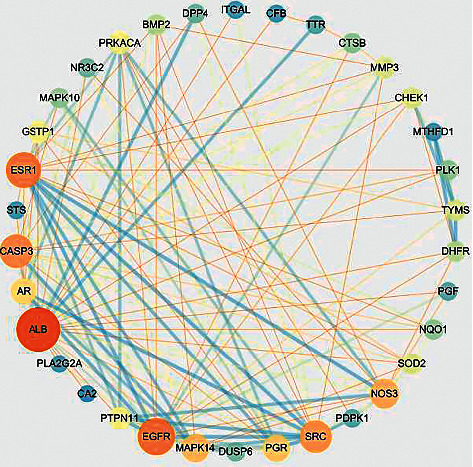
Protein interaction network of targets of bufalin on RCC. Blue represents the low degree, orange represents the high degree, and yellow represents the middle degree.

RCC and bufalin intersection targets were also added to the String database, and Cytoscape was utilized to create a functional protein-protein interaction network. The loop network contained 34 nodes that were linked and had 133 edges between them (Figure 3). The average local clustering coefficient was 0.714, and the average node degree was 7.82.

### 3.4. Enrichment Analysis of the Bufalin-RCC Target Network

#### 3.4.1. GO Enrichment Analysis

**Figure 4:**
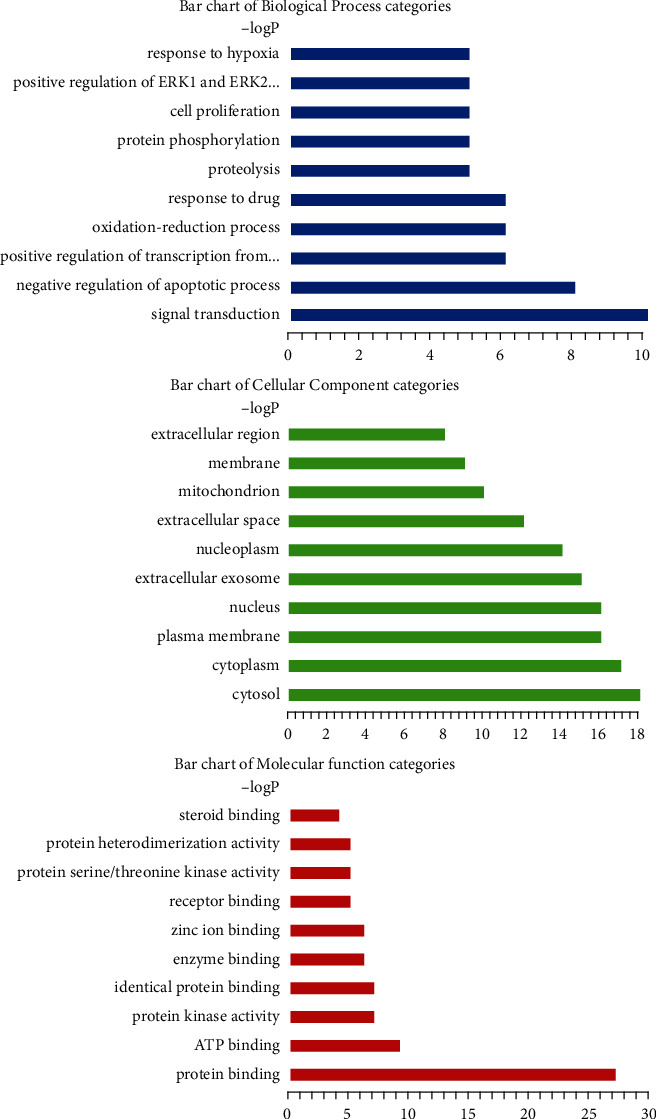
GO enrichment plot of bufalin resistance to RCC targets.

The 34-target information was entered into the David database for GO enrichment analysis. The results showed that 91 items including signal transduction, cell proliferation, protein phosphorylation, and others were related to biological processes. Further, 29 items including enzyme binding and others were related to molecular function. Similarly, 16 items including the nucleus, cytoplasm, and mitochondria were related to cell components.  Figure 4 and Table 2 show the top 10 BP, top 10 CC, and top 10 MF.

#### 3.4.2. Pathway of the Bufalin-RCC Network

**Figure 5:**
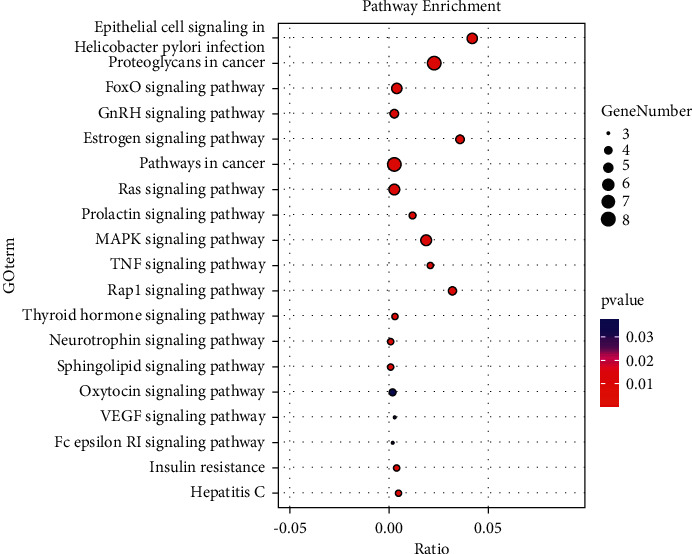
KEGG pathway enrichment diagram.

The information of 34 targets was imported into the David database for the KEGG pathway study. Ratio and *P* values were used to determine the degree of enrichment based on the number of target protein genes enriched in the pathway (Figure 5) [12]. After the analysis, 19 significant therapeutic pathways including epithelial cell signaling in *Helicobacter pylori* infection, proteoglycans in cancer, FoxO, GnRH, estrogen, pathways in cancer, Ras, prolactin, MAPK, TNF, Rap1, thyroid hormone, neurotrophin, sphingolipid, oxytocin, VEGF, Fc epsilon RI, insulin resistance, and hepatitis C were identified. In addition, four pathways including cancer pathways, the FoxO signaling pathway, the MAPK signaling route, and proteoglycans in cancer had more than five targets.

### 3.5. The Inhibitory Effect of Bufalin on Cells

The inhibitory effect of bufalin on human normal liver cells was not obvious. The cells were treated with bufalin (0.1, 1, and 10 *μ*M) for 24 hours, and the inhibitory rate was 0.2 ± 0.4%, 2.6 ± 0.6%, and 20.7 ± 0.5%, respectively. Therefore, we selected bufalin at a lower concentration for the next experiment.

Low concentrations of bufalin had no inhibitory effect on a kidney carcinoma cell line (Caki-1); however, the effect of bufalin on the Caki-1 cells was dramatically amplified with increasing time and concentration. When observed under a microscope, the membrane of the renal cell carcinoma cells displayed a bubble shape when bufalin acted on the Caki-1 cells, and some cells gradually changed from spindle-shaped to round and lifted off the dish.

**Figure 6:**
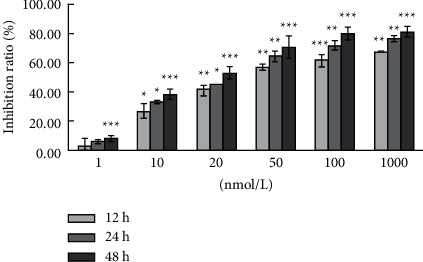
Bufalin inhibits the proliferation of Caki-1 cells.

To investigate the anticancer effects of bufalin on Caki-1 cells, the cells were treated with bufalin (0, 1, 10, 20, 50, 100, and 1000 nM) for 12, 24, or 48 h. The MTT assay indicated that bufalin had a significant time and dose-dependent inhibitory effect on Caki-1 cell proliferation. The IC_50_ values of bufalin on Caki-1 cells at 12, 24, and 48 h were 43.68 ± 4.63, 27.31 ± 2.32, and 18.06 ± 3.46 nM, respectively (Figure 6). The OD values measured at 12, 24, and 48 hours after bufalin acts on cells and the inhibition rates of different concentrations of bufalin at different times are described in the Supplementary Materials (available here).

### 3.6. Bufalin Induces Apoptosis in Caki-1 Cells

**Figure 7:**
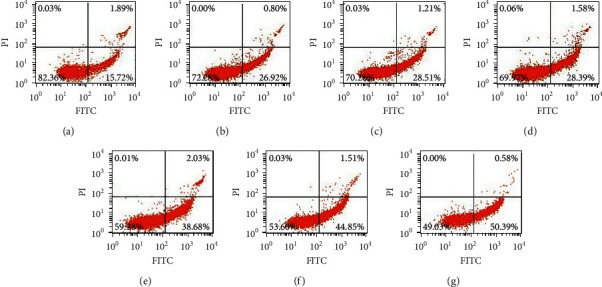
Bufalin induces apoptosis in Caki-1 cells (12 h): (a) Control; (b) 5 nmol/L; (c) 10 nmol/L; (d) 20 nmol/L; (e) 50 nmol/L; (f) 100 nmol/L; (g) 200 nmol/L.

**Figure 8:**
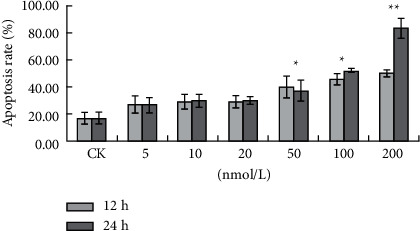
Bufalin induces apoptosis in Caki-1 cells (12 h and 24 h): (a) Control; (b) 5 nmol/L; (c) 10 nmol/L; (d) 20 nmol/L; (e) 50 nmol/L; (f) 100 nmol/L; (g) 200 nmol/L.

**Figure 9:**
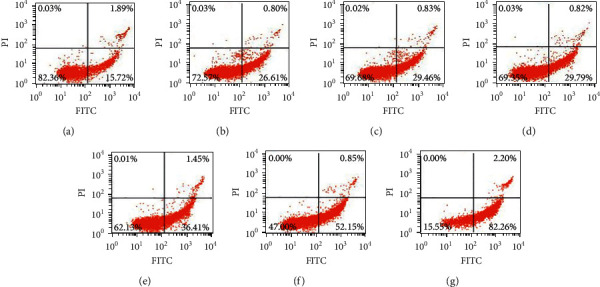
Bufalin induces apoptosis in Caki-1 cells (24 h): (a) Control; (b) 5 nmol/L; (c) 10 nmol/L; (d) 20 nmol/L; (e) 50 nmol/L; (f) 100 nmol/L; (g) 200 nmol/L.

The cells were treated with bufalin (0, 5, 10, 20, 50, 100, and 200 nM) for 12 or 24 hours, and flow cytometry was performed after Annexin V-PI labeling to see if the bufalin-induced reduction in cell survival was due to apoptosis. As shown, the percentage of apoptotic cells at 12 h was 17.61%, 27.72%, 29.71%, 29.97%, 40.71%, 46.36%, and 50.97%, respectively (Figures 7 and 8), and the values at 24 h were 17.61%, 27.41%, 30.29%, 30.61%, 37.86%, 53.00%, and 84.46%, respectively (Figures 8 and 9). These findings showed that bufalin induced apoptosis and inhibited cell viability in gastric cancer cells.

### 3.7. Bufalin Induces Cell Cycle Arrest at the G2/M Phase

**Figure 10:**
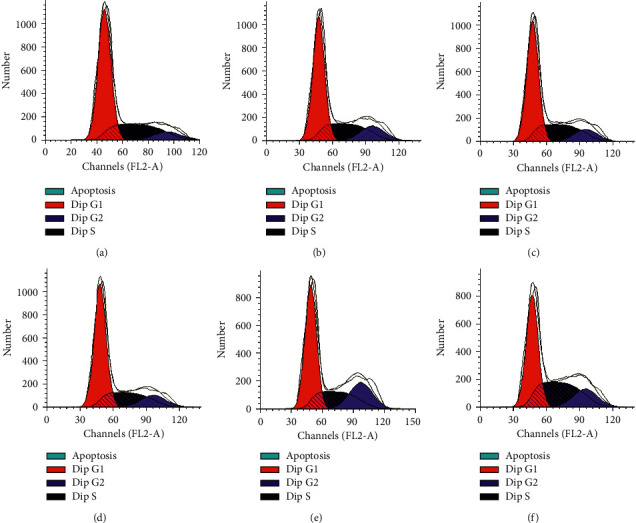
Bufalin induces cell cycle arrest in RCC Caki-1: (a) CK; (b) 1 nmol/L; (c) 5 nmol/L; (d) 10 nmol/L; (e) 20 nmol/L; (f) 50 nmol/L.

**Figure 11:**
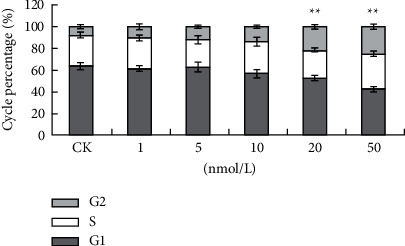
Bufalin induces cell cycle arrest in RCC Caki-1: (a) CK; (b) 1 nmol/L; (c) 5 nmol/L; (d) 10 nmol/L; (e) 20 nmol/L; (f) 50 nmol/L.

Flow cytometry was used to measure the number of Caki-1 cells in each phase to see if bufalin may cause cell cycle arrest. As evident from the results, bufalin increased the number of cells in the G2/M phase compared to the control treatment. The proportion of Caki-1 cells arrested in the G2/M phase increased sufficiently with increasing bufalin concentration, but the Caki-1 cell arrest proportion in the G2/M phase was significant in the 20 nmol/L and 50 nmol/L bufalin group. We also observed an arrest in the S phase with 50 nmol/L bufalin. These results show that bufalin inhibited Caki-1 cell growth by causing cell cycle arrest in the G2/M phase (Figures 10 and 11).

**Figure 12:**
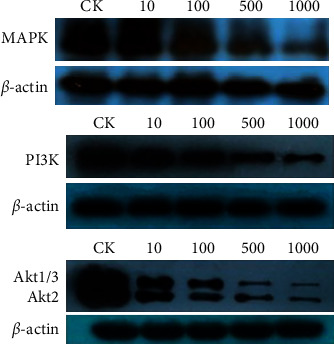
The expression of MAPK, P13K, and Akt protein after bufalin.

The expression levels of proteins implicated in the MAPK and PI3K/Akt signaling pathways were evaluated when Caki-1 cells were exposed to bufalin (10, 100, 500, and 1000 nM) for 24 hours to study the signal transduction route through which bufalin influences tumor growth. We observed that the level of MAPK and PI3K was decreased after 24 h in a dose-dependent manner, leading to a decrease in its downstream molecule p-Akt (Figure 12).

## 4. Discussion

Toxicity and resistance to currently available chemotherapeutic drugs are important barriers to cancer therapy. As a result, the ideal method would be to search for new and effective anticancer drugs with fewer side effects [13]. Bufalin is the main ingredient of the toad venom, causing cell death by inducing cell apoptosis and blocking the cell cycle in human cancer cells [14, 15]. In human liver cancer, prostate cancer, and multiple myeloma, bufalin has been shown to induce apoptosis and decrease proliferation [16–18]. However, it causes apoptosis and suppresses proliferation in Caki-1 cells via a molecular mechanism that is yet to be discovered. The study screened and discovered bufalin's therapeutic targets in RCC as well as the biological mechanisms that underpin them.

The experiments showed that bufalin exerted an antiproliferative effect on Caki-1 cells in a concentration-dependent manner. The cells underwent morphological changes, including reduced cell proliferation, circular shrinkage, and reduced adherence, which may be caused by the destruction of the cell membrane, cytoskeleton, and some internal cell structures by bufalin, resulting in significant morphological changes.

After treating Caki-1 cells with bufalin for 24 hours, the fraction of cells in the G0/G1 phase of the cell cycle was reduced significantly, while the fraction of cells in the G2/M phase continued to increase which is essentially consistent with the findings of Suehiro et al. and Ni et al. [10, 19]. This study also revealed that the number of cells in the G2/M phase increased as the bufalin concentration increased which is extremely significant, and when cycling cells face stress, they are arrested at one of the two cell cycle checkpoints, G2/M or G1/S.

Here, a “compound-target-pathway-disease” network was built using the network pharmacology approach to identify possible bufalin anti-RCC targets. Through KEGG pathway annotation and GO enrichment analysis, the target protein genes of bufalin contain anti-RCC including MAPK10 and PI3K. These target protein genes are involved in multiple tumor pathways and signal transduction pathways [20, 21]. In this study, the predicted potential targets were annotated through the KEGG pathway to obtain signal transduction pathways related to RCC, such as MAPK and PI3K signaling pathway. These pathways are linked to the development and occurrence of tumors.

One of the most significant routes in the eukaryotic signal transmission network is the mitogen-activated protein kinase (MAPK) chain, which is involved in gene expression control and cytoplasmic functional activities [22]. The MAPK pathway is one of the common intersections of signal transduction pathways, such as stress, inflammation, cell proliferation, differentiation, functional synchronization, transformation, and apoptosis, and the extracellular signal by the receptor, G/G of protein, protein kinase, and transcription factors, such as signal network, passed to the intracellular, involved in cell proliferation, differentiation, cancerous, transfer, and apoptosis [23]. In this study, we found that the levels of PI3K and MAPK were reduced after 24 hours in a dose-dependent manner. Therefore, we suggest that bufalin may inhibit the proliferation of Caki-1 cells by inhibiting the MAPK and PIK signaling pathways. These findings support bufalin's clinical use as an anticancer drug due to its therapeutic effects in renal cell carcinoma. However, we believe that more animal experiments are needed to better evaluate bufalin's therapeutic potential.

## 5. Conclusion

The possible targets of the bufalin on renal cell carcinoma were investigated using network pharmacological and experimental approaches. The bufalin-related therapeutic targets were identified via 35 intersecting targets. GO analysis identified 29 molecular functions, 16 cellular components, and 91 biological processes. KEGG pathway annotation identified 15 signal transduction pathways and 4 tumor-related pathways.

## Figures and Tables

**Table 1:** Target prediction of bufalin.

Pharma model	*Z*-score	Gene name	Gene ID	Pharma model	*Z*-score	Gene name	Gene ID
1j78_v	2.92521	GC	2638	2is7_v	1.00875	AKR1B1	231
1lho_v	2.78452	SHBG	6462	1j96_v	1.00818	AKR1C2	1646
2oi0_v	2.69038	Adam17	6868	2ywp_v	0.980715	CHEK1	1111
1ov4_v	2.31158	SULT2A1	6822	2p3g_v	0.944171	MAPKAPK2	9261
1a28_v	2.30115	PGR	5241	1kbo_v	0.92543	NQO1	1728
2pix_v	1.82985	AR	367	1ya3_v	0.887381	NR3C2	4306
1xdd_v	1.34837	ITGAL	3683	1gfw_v	0.853259	CASP3	836
1ydr_v	1.29681	PRKACA	282322	1csb_v	0.828267	CTSB	1508
1pmv_v	1.25964	MAPK10	5602	2h8h_v	0.822228	SRC	6714
2rku_v	1.1589	PLK1	5347	2zas_v	0.819623	ESRRG	2104
1e7e_v	1.15408	ALB	213	1m9r_v	0.817268	NOS3	4846
2qo8_v	1.15252	CA2	760	1n83_v	0.80888	RORA	6095
2oht_v	1.14078	BACE1	23621	2ocf_v	0.693666	ESR1	2099
1p49_v	1.08482	STS	412	1reu_v	0.668496	BMP2	650
2j7x_v	1.0527	Esr2	25149	1l6l_v	0.529143	APOA2	336

**Table 2:** The findings of the GO analysis, which comprised the top 10 MF, top 10 CC, and top 10 BP.

GO term	Gene ratio	Ontology
Signal transduction	10	BP
Negative regulation of the apoptotic process	8	BP
Positive regulation of transcription from RNA polymerase II promoter	6	BP
Oxidation-reduction process	6	BP
Response to drug	6	BP
Proteolysis	5	BP
Protein phosphorylation	5	BP
Cell proliferation	5	BP
Positive regulation of ERK1 and ERK2 cascade	5	BP
Response to hypoxia	5	BP
Cytosol	18	CC
Cytoplasm	17	CC
Plasma membrane	16	CC
Nucleus	16	CC
Extracellular exosome	15	CC
Nucleoplasm	14	CC
Extracellular space	12	CC
Mitochondrion	10	CC
Membrane	9	CC
Extracellular region	8	CC
Protein binding	27	MF
ATP binding	9	MF
Protein kinase activity	7	MF
Identical protein binding	7	MF
Enzyme binding	6	MF
Zinc ion binding	6	MF
Receptor binding	5	MF
Protein serine/threonine kinase activity	5	MF
Protein heterodimerization activity	5	MF
Steroid binding	4	MF

## Data Availability

The data used to support the findings of this study are included with the Supplementary Information files.

## Supplementary Materials

Supplementary Materials The number of Caki-1 cells was significantly changed after treatment with bufalin. Results indicated that bufalin can inhibit the proliferation of Caki-1 cells with a time- and dose-dependent manner (Table 1 and Figure 1). The OD values measured at 12, 24, and 48 hours after bufalin acts on cells and the inhibition rates of different concentrations of bufalin at different times are described (Table 2). ()
