# Supplementary material for: Therapeutic Targets of Bufalin on Renal Carcinoma and Mechanisms: Experimental Validation of Network Pharmacology Analysis
Source: Evid Based Complement Alternat Med. 2022 Jan 28;2022:5469795. doi: 10.1155/2022/5469795 (PMC8816594; doi:10.1155/2022/5469795)
Supplement: Supplementary Materials — The number of Caki-1 cells was significantly changed after treatment with bufalin. Results indicated that bufalin can inhibit the proliferation of Caki-1 cells with a time- and dose-dependent manner (Table 1 and Figure 1). The OD values measured at 12, 24, and 48 hours after bufalin acts on cells and the inhibition rates of different concentrations of bufalin at different times are described (Table 2). () [file 5469795.f1.pdf]

Table 1 Growth curve test results in Bufalin treatment of RCC Caki-1

Bufalin	Cell Population($\bar{X}\pm S/1\times10^4$ 个， n=3)						
(nmol/L)	1d	2d	3d	4d	5d	6d	7d
CK	1.225±0.0714	1.294±0.0509	3.497±0.0400	4.607±0.0569	4.733±0.0445	4.776±0.0632	4.912±0.0571
1	1.204±0.0899	1.285±0.0379	3.523±0.0356	4.525±0.0126	4.637±0.0033	4.727±0.0111	4.803±0.0341
10	1.118±0.0714	1.231±0.0222	3.122±0.0351	4.082±0.0393	4.118±0.0508	4.218±0.0526	4.201±0.0534
20	1.022±0.0117	1.011±0.0539	2.120±0.0561	2.205±0.0483	2.525±0.0587	2.680±0.0531	2.795±0.0393
50	0.940±0.0404	1.000±0.0628	0.917±0.0482	0.791±0.0543	0.710±0.0594	0.692±0.0382	0.626±0.0415
100	0.873±0.0520	0.899±0.0481	0.815±0.0414	0.699±0.0294	0.595±0.0583	0.507±0.0312	0.361±0.0575
1000	0.779±0.0417	0.697±0.0429	0.514±0.0583	0.471±0.0567	0.340±0.0511	0.256±0.0574	0.156±0.0545

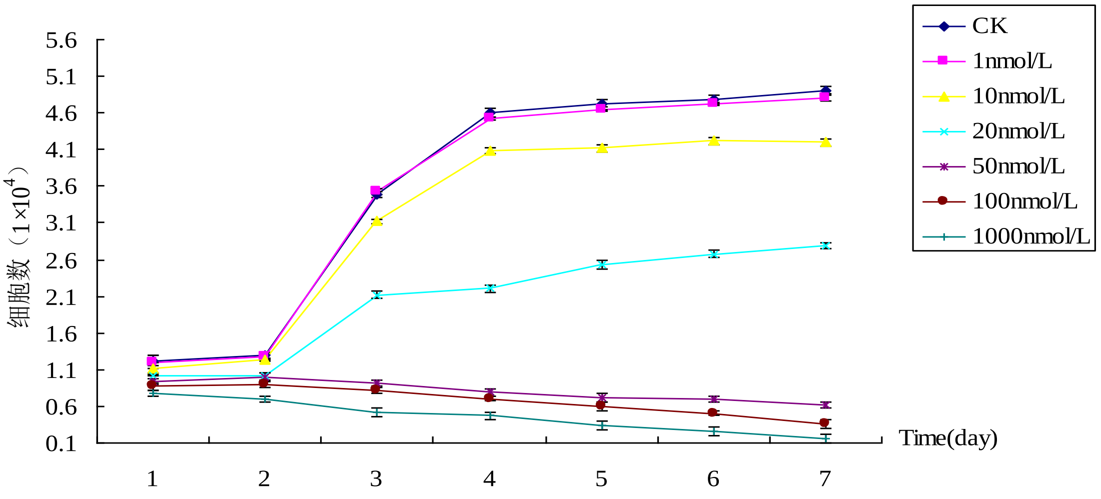

Fig. 1 Growth curve of RCC Caki-1 before and after treatment with Bufalin

Table 2 The OD values of RCC Caki-1after Bufalin($\bar{X}\pm S$， n=3)

Bufalin (nmol/L)	12h	24h	48h
1	1.179±0.0537	1.247±0.0130	1.252±0.0180
10	0.887±0.0509	0.888±0.0102	0.844±0.0319
20	0.713±0.0366	0.725±0.0008	0.639±0.0451
50	0.527±0.0214	0.474±0.0339	0.403±0.0758
100	0.472±0.0426	0.383±0.0331	0.275±0.0439
1000	0.402±0.0058	0.320±0.0189	0.259±0.0383
CK	1.215±0.0783	1.326±0.1699	1.359±0.0171
